# Preincubation with a low-dose hydrogen peroxide enhances anti-oxidative stress ability of BMSCs

**DOI:** 10.1186/s13018-020-01916-y

**Published:** 2020-09-09

**Authors:** Lei Wang, Fei Zhang, Wuxun Peng, Jian Zhang, Wentao Dong, Dajiang Yuan, Zhenwen Wang, Yinggang Zheng

**Affiliations:** 1 Department of Orthopedics, The Affliated Hospital of Guizhou Medical University, Guiyang, 550004 Guizhou China; 2 Guizhou Medical University, Guiyang, 550004 Guizhou China

**Keywords:** Bone marrow mesenchymal stem cells, Oxidative stress injury, Pretreatment, Hydrogen peroxide, Apoptosis

## Abstract

**Objective:**

To investigate the effects of low-concentration hydrogen peroxide pretreatment on the anti-oxidative stress of the bone marrow mesenchymal stem cells (BMSCs).

**Methods:**

Rabbit BMSCs were isolated and cultured by density gradient centrifugation combined with the adherence method. Then, the third generation of well-grown BMSCs was continuously treated with 50-μM hydrogen peroxide (H_2_O_2_) for 8 h as the optimal pretreatment concentration and the BMSCs were continuously applied for 24 h with 500 μM H_2_O_2_, and the optimal damage concentration was determined as the oxidative stress cell model. The experiment was divided into three groups: control group, high-concentration H_2_O_2_ injury group (500 μM), and low-concentration H_2_O_2_ pretreatment group (50 μM + 500 μM). In each group, the DCFH-DA fluorescence probe was used to detect the reactive oxygen species (ROS). ELISA was used to detect the activity of superoxide dismutase (SOD) and catalase (CAT), and the TBA method was used to detect malondialdehyde (MDA). The mitochondrial membrane potential was detected by JC-1. The cell viability was detected by CCK-8 method, while flow cytometry and TUNEL/DAPI double staining were performed to detect cell apoptosis. Hence, the effect of H_2_O_2_ pretreatment on the anti-oxidative stress of BMSCs was investigated. One-way analysis of variance was performed using SPSS 19.0 statistical software, and *P* < 0.05 was considered statistically significant.

**Results:**

A large number of typical BMSCs were obtained by density gradient centrifugation and adherent culture. The oxidative stress cell model was successfully established by 500-μM H_2_O_2_. Compared with the high-concentration H_2_O_2_ injury group, the low-concentration H_2_O_2_ pretreatment reduced the production of ROS [(62.33 ± 5.05), *P* < 0.05], SOD and CAT activities significantly increased (*P* < 0.05), and MDA levels significantly decreased (*P* < 0.05). The mitochondrial membrane potential fluorescence changes, the ratio of red/green fluorescence intensity of the high-concentration H_2_O_2_ injury group was less, and the ratio of the low-concentration H_2_O_2_ pretreatment group was significantly higher than that. The ratio of red/green increased by about 1.8 times (*P* < 0.05). The cell viability and survival rate of BMSCs were significantly increased in low-concentration H_2_O_2_ pretreatment group (*P* < 0.05), and the cell apoptosis rate was significantly decreased (*P* < 0.05).

**Conclusion:**

Pretreatment with low-concentration H_2_O_2_ can enhance the anti-oxidative stress ability and reduce their apoptosis of BMSCs under oxidative stress.

## Background

BMSCs are pluripotent stem cells that have the potential for multi-directional differentiation [1]. In recent years, due to its high proliferative capacity and high differentiation ability, it has attracted widespread attention in stem cell research. At present, the use of BMSC transplantation for the treatment of injury has been proven to be a very promising [2–4]. However, clinical practice and experiments have shown that ischemia, hypoxia, and inflammatory cell infiltration in the injured area produce a large amount of ROS, forming an oxidative stress microenvironment. This results in the death of most of the transplanted BMSCs within 24 h after transplantation, badly affecting the transplantation [5]. How to improve the survival rate of cells after transplantation is an urgent problem to be solved in clinical research and application of stem cells.

Oxidative stress is the key cause of death of transplanted BMSCs [6]. The important mechanism is that the oxidative stress microenvironment in the osteonecrosis area increases the production of ROS and/or decreases the ability of scavenging ROS, resulting in an imbalance between the production and clearance of ROS [7, 8]. Excessive ROS can increase mitochondrial membrane permeability, mitochondrial swelling, mitochondrial permeability (mMPTP) opening, and mitochondrial DNA (mtDNA) damage. This leads to dysfunction of mitochondrial electron transport chain, disturbance of tricarboxylic acid cycle, disturbance of ATP synthesis, decrease of transmembrane potential, release of cytochrome C, activation of Caspase-9, formation of caspase-dependent apoptosis pathway, and final induction of BMSCs apoptosis [9]. Therefore, determining how to eliminate excessive ROS to enhance the survival and anti-apoptosis ability of transplanted stem cells is the key to improve the effective utilization of stem cells and enhance the therapeutic effect.

Adaptive cytoprotection refers to the application of “sub-traumatic” factors or “survival-promoting” factors to stimulate cells in advance in order to reduce the damage to cells caused by subsequent more serious damage factors [10]. Therefore, We hypothesized that oxidative stress pretreatment of BMSCs can enhance its anti-oxidant stress, thereby enhancing the survival and proliferation of BMSCs in the necrotic zone, thus improving the therapeutic effect of transplantation. The present study established an oxidative stress cell model by H_2_O_2_, and BMSCs were treated with the best pretreatment concentration and the best damage concentration and observed the survival of BMSCs under different concentrations of H_2_O_2_. Furthermore, the effect of low-concentration hydrogen peroxide pretreatment on the anti-oxidative stress ability of BMSCs was evaluated, providing a theoretical basis for improving the efficacy of BMSC transplantation.

## Materials and methods

### Animals

In the present study, 4–6-week-old specific pathogen-free (SPF) male New Zealand white rabbits (1.0–2.0 kg) were provided by the Animal Research Center of Guizhou Medical University.

### Instruments and reagents

Desktop high-speed refrigerated centrifuge (Allegra 64R High Speed Centrifuge, Beckman, USA); flow cytometry (Navios; Beckman, USA); multi-function microplate reader (Synergy; Biotech, USA); inverted fluorescence microscope (DM1LLED; Leica, Germany); laser confocal microscopy (LSM710; ZEISS, Germany); DMEM medium, fetal bovine serum, and trypsin (Gibco, New Zealand); H_2_O_2_ (Chengdu Jinshan Chemical Reagent Co., Ltd., China); CCK-8 solution (Japan Tongren Chemical Research Institute, China); annexin V-FITC/PI double-stained cell apoptosis detection kit (BD; San Diego, USA); intracellular ROS detection kit (Sigma, USA); JC-1 cell mitochondrial membrane potential detection kit and TUNEL apoptosis detection kit (Nanjing Kaiji Biotechnology Co., Ltd., China); DAPI and BCA protein assay kit (Beijing Suobao Biotechnology Co., Ltd., China); superoxide disproportionation enzyme detection kit and catalase detection kit (Nanjing, China); and malondialdehyde detection kit (Shanghai Biyuntian Biotechnology Co., Ltd., China).

### Isolation and culture of rabbit BMSCs

In the present study, 4–6-week-old New Zealand white rabbits were used. Under sterile conditions, the bone marrow fluid was taken from the distal femur and proximal humerus, and the cells were separated by density gradient centrifugation. Then, these were incubated in a cell incubator (37 °C, 5% CO_2_). When the primary cells reached 80–90% of confluence, these are passaged at a ratio of 1:3. Cells passaged up to the 3rd generation were used for the experimental treatment.

### Establishment of the oxidative stress model for BMSCs

The 3rd generation BMSCs were divided into three groups: control group, high-concentration H_2_O_2_ injury group (500 μM), and low-concentration H_2_O_2_ pretreatment group (50 μM + 500 μM). When cell confluence reached approximately 80%, according to the experimental group, the low-concentration H_2_O_2_ pretreatment group was first treated with 50 μM of H_2_O_2_ for 8 h, recovered for 12 h, and finally treated with 500 μM of H_2_O_2_ for 24 h, the high-concentration H_2_O_2_ injury group was directly treated with 500 μM of H_2_O_2_ for 24 h, and the control group was routinely cultured.

### Detection of intracellular ROS

The detection of cellular ROS was carried out using the fluorescent probe DCFH-DA, which can be oxidized to the progesterone high-fluorescent compound dichlorofluorescein (DCF). Then, these cells were incubated with DCFH-DA for 30 min at 37 °C and washed twice with phosphate-buffered saline (PBS). The fluorescence intensity was observed using a laser confocal microscope. Five fields were randomly selected under a high-power microscope, and the fluorescence intensity was calculated by fluorescence expression.

#### Evaluation of SOD, MDA, and CAT activity

After cells are treated with H_2_O_2_, these cells were digested with trypsin and centrifuged at 1000 rpm for 10 min, and the cell pellet was collected. Then, these cells were disrupted by ultrasonography, centrifuge for 10 min at 12,000 rpm, and the supernatant was taken for the experiment. Each indicator was tested according to kit instructions.

### Detection of cell activity by CCK-8

After cells are treated with H_2_O_2_, these cells were washed three times with PBS, and 100 μL of a complete medium and 10 μL of CCK-8 solution were added to each well. After incubation for 2 h, the enzyme-linked immunosorbent assay was performed at 450 nm. Then, the absorbance value (OD) was measured.

### Annexin V-FITC/PI-detected apoptosis

The H_2_O_2_-treated cells were washed twice with PBS, and these cells were resuspended in binding buffer. Then, 100 μL of the cell suspension was transferred to a 5-ml centrifuge tube, and 5 μL of FITC and 5 μL of propidium iodide (PI) were added. Next, these cells were gently vortexed and incubated for 15 min in the dark at room temperature (25 °C). Afterwards, 400 μL of binding buffer was added to each tube and analyzed by flow cytometry.

### TUNEL and DAPI double staining detection of cell apoptosis

Cells were washed with PBS, fixed in 4% formaldehyde solution for 25 min at 4 °C, and washed with PBS for 5 min, for three times. Then, 1% Triton X-100 was incubated for 5 min at room temperature and washed five times with PBS for three times. Afterwards, 50 μL of equilibration buffer was added to each group and equilibrated for 10 min at room temperature. Subsequently, cells were incubated with 50 μL of TdT working solution for 60 min in the dark at 37 °C. Five fields were randomly selected for the high-power microscope for apoptotic cell counting, and each experiment group was repeated at least three times.

### Observation of mitochondrial membrane potential by JC-1

JC-1 was prepared according to the manufacturer’s instructions. Cells were incubated with the stains for 30 min at 37 °C and washed three times with PBS. Finally, the fluorescence was observed under a laser confocal microscope. Five fields were randomly selected under a high-power microscope, and the red/green fluorescence intensity ratio was calculated. Each experiment group was calculated for at least three times.

### Statistical methods

All data were analyzed using SPSS 19.0 statistical software. Measurement data were expressed as mean ± standard deviation ($\overline{x}$ ± SD). One-way analysis of variance was used for multiple comparisons. The LSD *t* test was used for comparisons between groups. *P* < 0.05 was considered statistically significant.

## Results

### Changes in cell morphology after H_2_O_2_ induction

**Fig. 1:**
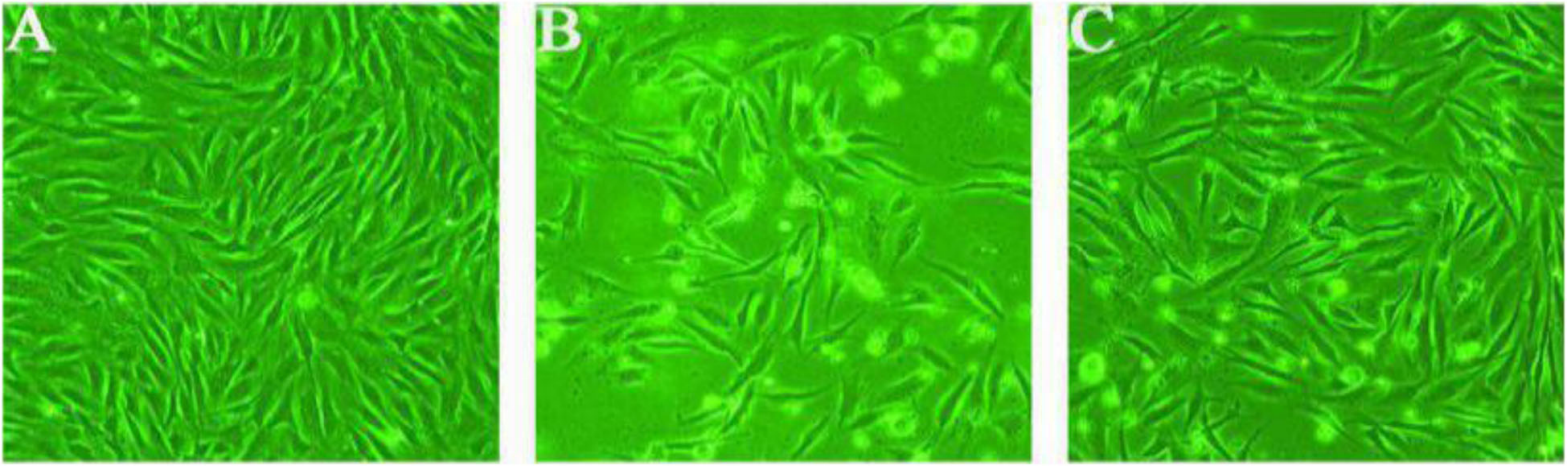
Observation of cell morphology using an inverted phase-contrast microscope. **a** Control group (×100); **b** High-concentration H_2_O_2_ injury group (×100); **c** Low-concentration H_2_O_2_ pretreatment group (×100)

Cells not treated with H_2_O_2_ had normal cell morphology, were spindle-shaped, and had few apoptotic cells (Fig. 1a). Cells in the high-concentration H_2_O_2_ treatment group became smaller, had a long spindle shape, and the number of apoptotic cells significantly increased (Fig. 1b). However, the morphology of cells in the low-concentration H_2_O_2_ pretreatment group did not significantly change, and the apoptosis was significantly reduced (Fig. 1c).

### Intracellular ROS

**Fig. 2:**
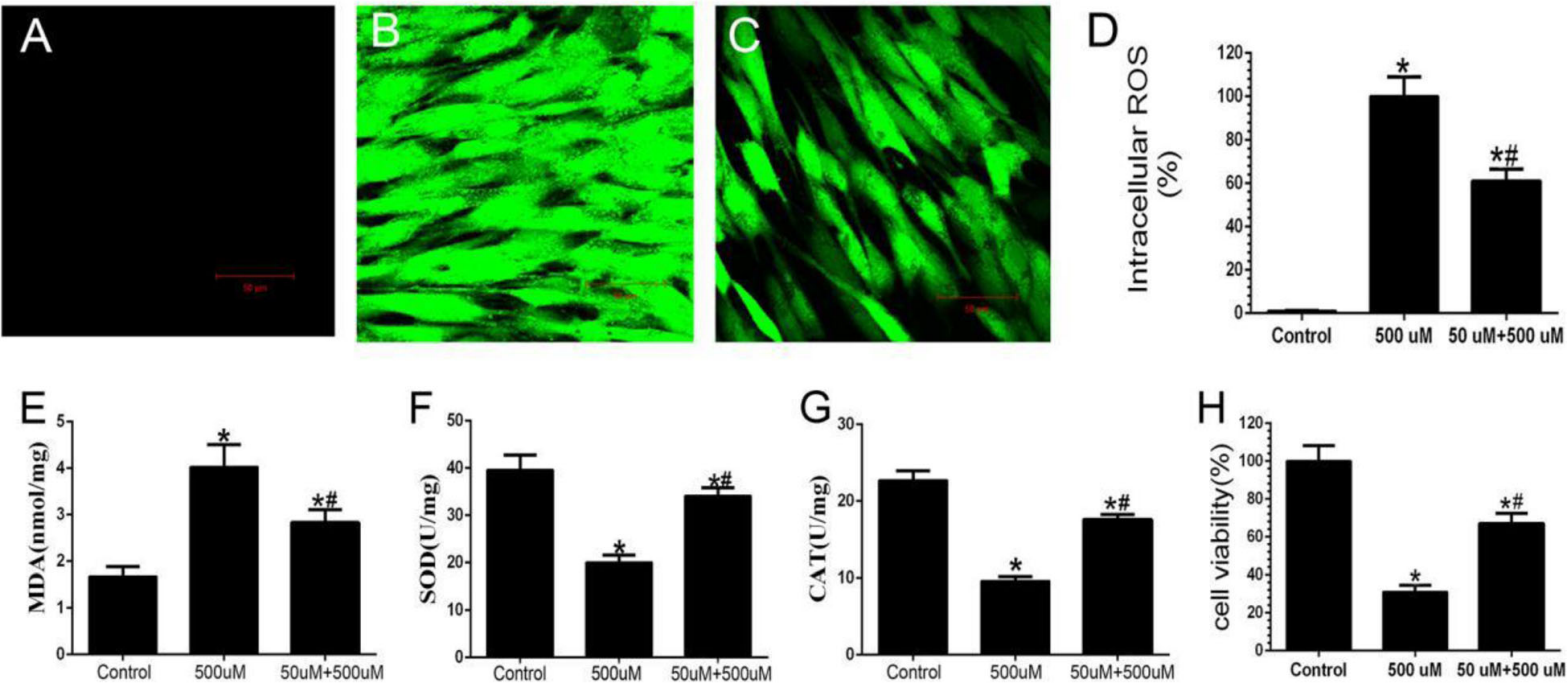
Detection of intracellular ROS, MDA, SOD, CAT, and cell viability. **a**–**c** Detection of ROS, control group, high-concentration H_2_O_2_ injury group and Low-concentration H_2_O_2_ pretreatment group respectively (×100); **d** Quantitative analysis of DCF fluorescence intensity; **e** Intracellular MDA content; **f** Intracellular SOD viability; **g** Intracellular CAT viability; **h** Cell viability; *n* = 5; Compared with the control group, ^*^P < 0.05; Compared with the high-concentration H_2_O_2_ injury group, ^#^*P* < 0.05

The changes in absorption value and fluorescence intensity of each group were detected by a fluorescence microscope. The control group was not treated with H_2_O_2_, there was merely a very small amount of ROS in cells, and the content of ROS is (4.53 ± 0.49) (Fig. 2a). Compared with the control group, treatment with 500 μM of H_2_O_2_ for 24 h significantly increased ROS levels, and the content of ROS was (88.3 ± 7.67), which showed strong green fluorescence (Fig. 2b). It is suggested that excessive ROS is produced after being treated with a high concentration of H_2_O_2_. However, after pretreatment with 50 μM of H_2_O_2_ for 8 h, these intracellular ROS levels were effectively reduced, (62.33 ± 5.05) (Fig. 2c), and the difference was statistically significant (Fig. 2d).

### SOD, MDA, CAT, and cell viability

The experimental results show that MDA in the pretreatment group was 2.83 ± 0.28 nmol/mg (Fig. 2e), while SOD, CAT, and cell viability were 34.11 ± 1.72 U/mg, 17.59 ± 0.68 U/mg, and 77.08 ± 4.88%, respectively (Figs. 2f–h). Compared with non-pretreatment, MDA content significantly decreased, SOD, CAT, and cell viability increased. These results suggest that 50-μM H_2_O_2_ pretreatment can improve the oxidative damage of cells by increasing the activities of endogenous antioxidant enzymes.

### Intracellular mitochondrial membrane potential

**Fig. 3:**
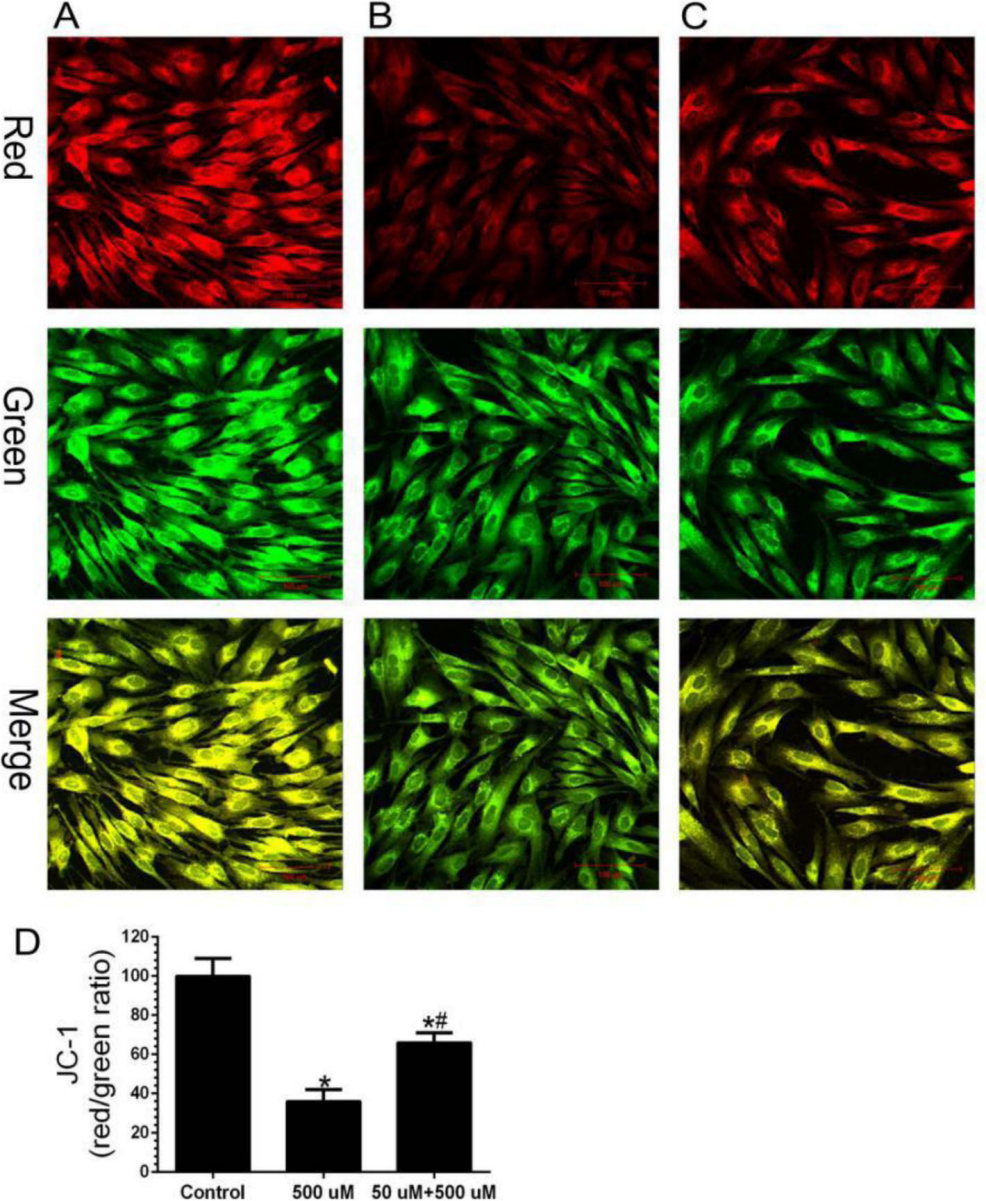
Detection of mitochondrial membrane potential by JC-1. **a** Control group (×400); **b** High-concentration H_2_O_2_ injury group (×400). **c** Low-concentration H_2_O_2_ pretreatment group (×400). **d** Quantitative analysis of membrane potential changes; *n* = 3; Compared with the control group, ^*^P < 0.05; Compared with the high-concentration H_2_O_2_ injury group, ^#^*P* < 0.05

The protective effect of low-concentration H_2_O_2_ pretreatment on mitochondria could be observed by JC-1 staining. Normal cells revealed high red and high green fluorescence (Fig. 3a). However, when cells were exposed to high concentrations of H_2_O_2_ (500 μM) for 24 h, Δψm rapidly depolarized, the green fluorescence increased, and the red fluorescence decreased, the ratio of red to green is 36.34 ± 5.62% (Fig. 3b). After the 50-μM H_2_O_2_ pretreatment, the green fluorescence intensity was reduced and red fluorescence was restored, and the ratio of red to green is 66.52 ± 4.71% (Fig. 3c). The pretreatment group was 1.8 times higher than the non-pretreatment group (Fig. 3d). This suggests that low-concentration H_2_O_2_ pretreatment can achieve anti-apoptotic effects by inhibiting the mitochondrial damage pathway.

### Annexin-V/PI double staining was used to detect apoptosis

**Fig. 4:**
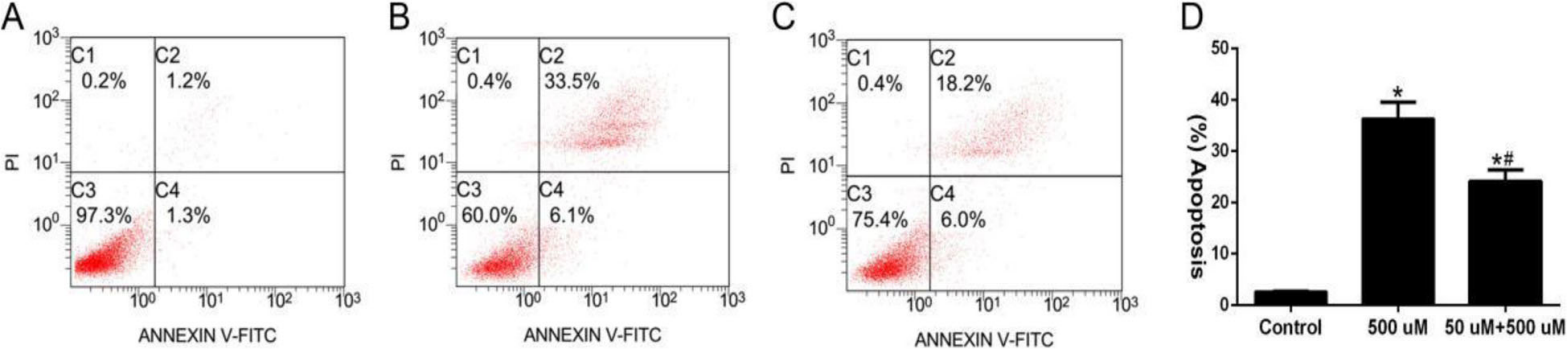
Apoptosis detected by annexin-V/PI staining. **a** Control group (×400); **b** High-concentration H_2_O_2_ injury group (×400); **c** Low-concentration H_2_O_2_ pretreatment group (×400); **d** Quantitative analysis of cell apoptosis; *n* = 3; Compared with the control group, ^*^P < 0.05; Compared with the high-concentration H_2_O_2_ injury group, ^#^*P* < 0.05

The annexin V/PI apoptosis assay revealed that the apoptotic rate was 2.56 ± 0.17% in the blank group (Fig. 4a) and 39.67 ± 3.20% in the non-pretreatment group (Fig. 4b). Compared with non-pretreatment, the apoptosis rate in the pretreatment group significantly decreased, 24.26 ± 2.10% (Fig. 4c), and the difference was statistically significant (Fig. 4d). Hence, a low-concentration H_2_O_2_ pretreatment reduces apoptosis and promotes cell survival under oxidative stress.

### TUNEL/DAPI detects apoptosis

**Fig. 5:**
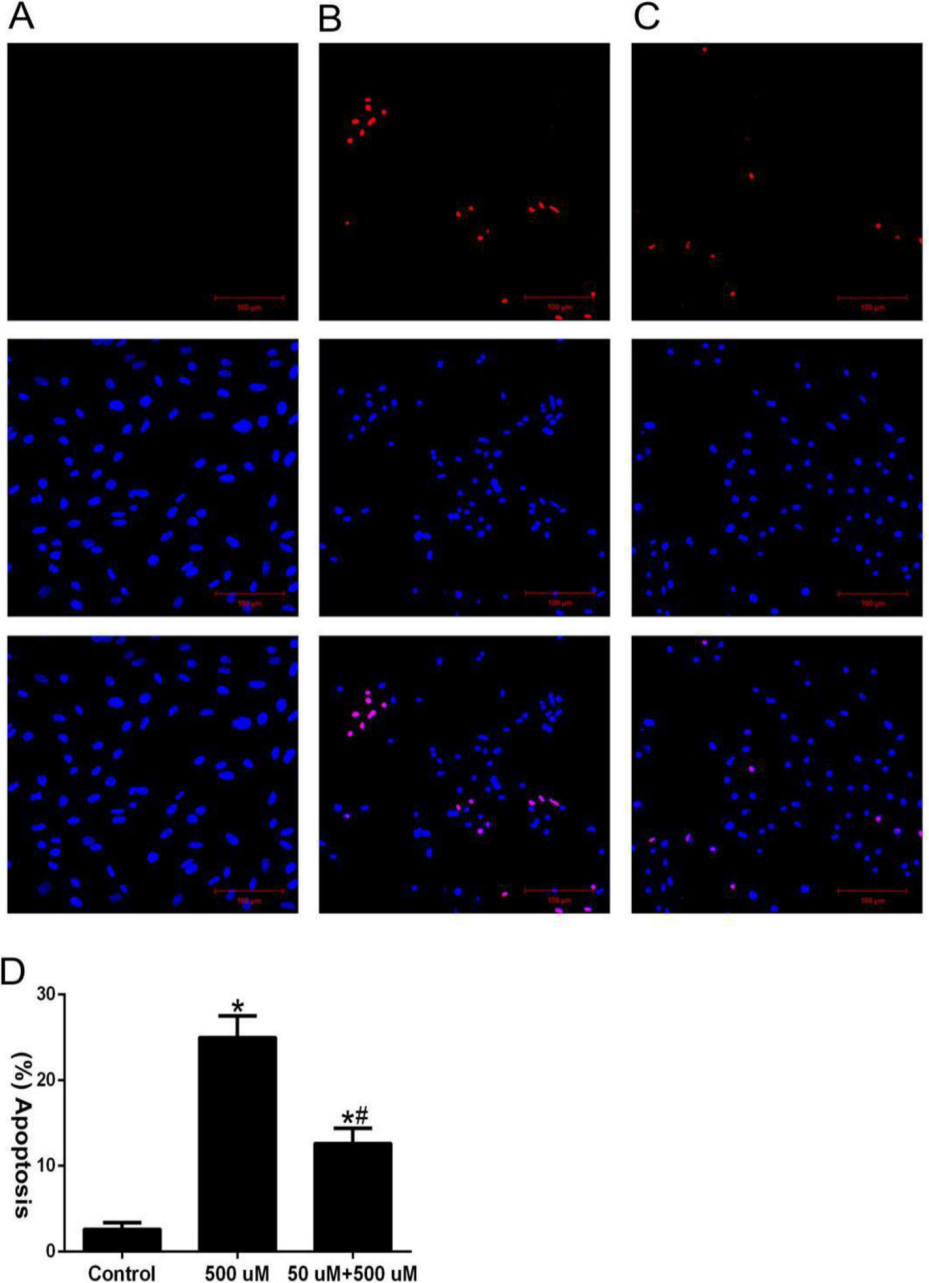
Apoptosis detected by TUNEL/DAPI staining. **a** Control group (×400); **b** High-concentration H_2_O_2_ injury group (×400); **c** Low-concentration H_2_O_2_ pretreatment group (×400); **d** Quantitative analysis of cell apoptosis; *n* = 3; Compared with the control group, ^*^P < 0.05; Compared with the high-concentration H_2_O_2_ injury group, ^#^*P* < 0.05

The results of apoptosis detected by TUNEL/DAPI method showed that compared with the control group (Fig. 5a), the proportion of TUNEL-positive cells in the high-concentration H_2_O_2_ injury group significantly increased, and the apoptosis was 25.72 ± 2.33% (Fig. 5b). However, the proportion of TUNEL-positive cells decreased in the low-concentration H_2_O_2_ pretreatment group, and the apoptosis rate was 12.67 ± 0.72% (Fig. 5c). Compared with the non-pretreatment group, the apoptosis rate of the pretreatment group decreased by approximately 1/2 (Fig. 5b). These results suggest that pretreatment can significantly reduce BMSC apoptosis under oxidative stress.

## Discussions

The ischemic and hypoxic microenvironment in osteonecrosis area makes cells suffer strong oxidative stress, resulting in changes and loss of cell function, inhibition of cell proliferation, and apoptosis, which seriously affects the effect of cell therapy [11, 12]. Studies have shown that the oxidative stress microenvironment in the ischemic necrotic area of the femoral head causes the grafted BMSCs to suffer oxidative stress, causes a large amount of ROS, in BMSCs to induce premature senescence and apoptosis of BMSCs, and decreases the proliferation and osteogenic differentiation of surviving BMSCs, which seriously affects the repair effect of the tissue-engineered bone on ANFH [13]. Strengthening the anti-oxidant stress ability of BMSCs in the transplantation area is the key to improve the curative effect of transplantation.

Numerous studies have shown that pre-repeated short-term low-intensity damage stimulation can induce cells or the body to tolerate the subsequently sustained similar high-intensity damage stimulation, which is a ubiquitous adaptive protection phenomenon. Liu and other studies [14] have shown that after myocardial infarction in rats, the transplantation of cardiac progenitor cells (CPCs) and the H_2_O_2_ pretreatment of CPCs can significantly improve the survival rate of CPCs, improve ventricular systolic function, and improve efficacy. Li and other reports [15] on diabetic cardiomyopathy, high glucose, and the H_2_O_2_ pretreatment of mesenchymal stem cells (MSCs) can significantly improve the survival, proliferation, and angiogenesis of MSCs and promote cardiac repair. Therefore, in this study, we used the oxidative stress model of BMSCs induced by H_2_O_2_. BMSCs were pretreated with a low concentration of H_2_O_2_ at first, and then, the effect was observed under the condition of a high concentration of H_2_O_2_. It was found that pretreatment with a low concentration of H_2_O_2_ could significantly improve the resistance of BMSCs to oxidative stress.

As our experimental results show, after treating BMSCs with high concentrations of H_2_O_2_, the flow cytometry revealed that the apoptotic rate significantly increased after treatment with high concentrations of H_2_O_2,_ and the apoptotic rate was 39.67 ± 3.20%, and cell viability was 31.13 ± 2.11%. Furthermore, DAPI staining revealed that some of the nucleus contractions became smaller, and the nucleoplasm was densely stained or fragmented. This indicates that these cells were obviously damaged, suggesting that the oxidative stress environment was not conducive to the survival of BMSCs. However, when cells were pretreated with 50-μM low-concentration H_2_O_2_ for 8 h, cell viability significantly increased to 67.09 ± 5.38%, and the apoptosis rate was 24.26 ± 2.10% in the pretreatment group. These results show that apoptosis was significantly lower than that in the high-concentration injury group, and the cell viability rate increased significantly. This confirms that low-concentration H_2_O_2_ pretreatment could significantly improve the cell survival of BMSCs after oxidative stress injury and inhibit the apoptosis.

The occurrence of apoptosis is a complex process that involves multiple factors, ROS production, mitochondrial depolarization, chromatin agglutination, and nuclear fission [16]. ROS is a general term for oxygen-containing free radicals and free radical-forming peroxides associated with oxygen metabolism in living organisms, and the oxidizing properties are very active [17]. ROS can cause membrane lipid peroxidation, protein cross-linking and degradation, deoxyribonucleic acid cleavage, and the mitochondrial dysfunction of BMSCs [18]. Under physiological conditions, the body continuously produces active oxygen, and the body’s antioxidant system continuously removes active oxygen, which is in a dynamic equilibrium, and does not damage the body [19]. However, when harmful stimuli occur, a large amount of active oxygen is generated, and the antioxidant system has limited the ability to remove these, eventually leading to oxidative damage [20]. The main systems for scavenging ROS in cells are antioxidant enzymes such as SOD and CAT, the intracellular levels of both of them can represent the changes of intracellular anti-oxidant capacity to a certain extent. The present experiment revealed that pretreatment with a low concentration of H_2_O_2_ can significantly reduce the production of intracellular ROS and reduce cellular oxidative stress damage. The results show that the activities of SOD and CAT in a low-concentration H_2_O_2_ pretreatment group were significantly higher than those in a high-concentration H_2_O_2_ injury group, and the contents of MDA and ROS decreased significantly, suggesting that H_2_O_2_ pretreatment may increase the activity of SOD and CAT, so as to enhance the resistance to oxidative damage.

Mitochondria are the energy and metabolic centers of eukaryotes and are also organelles that play a key regulatory role in apoptosis and signaling [21]. The mitochondrial membrane potential declined as a preliminary event of apoptosis. Oxidative stress injury leads to apoptosis through the mitochondrial pathway, which destroys the stability of the mitochondrial membrane, causes cytochrome C to be released from the mitochondria to the cytoplasm, then binds to the apoptotic protein activator in the cytoplasm, and leads to apoptosis by proteolysis [22]. In this study, the mitochondrial membrane potential of cells was detected using JC-1 method, and damage to the mitochondrial membrane potential induced by H_2_O_2_ was observed [23]. When the mitochondrial membrane potential becomes high, JC-1 forms a polymer that produces red fluorescence, while when the mitochondrial membrane potential is low, it produces a green fluorescence. This allows changes in the mitochondrial membrane potential to be detected by fluorescence color transitions [24, 25]. In our results, the red fluorescence in the high-concentration H_2_O_2_ injury group could be observed, the green fluorescence was significantly lower, and the ratio of red to green is 36.34 ± 5.62%, indicating that H_2_O_2_ can decrease the mitochondrial membrane potential. Furthermore, the red-green ratio in the pretreatment group significantly increased, the ratio is 66.52 ± 4.71%, indicating that these cells maintained a high membrane potential level. These results show that low-concentration H_2_O_2_ pretreatment can maintain the mitochondrial membrane potential level and protect mitochondrial function to some extent.

The limitations of the present study include the following, although this experimental study proved that oxidative stress preconditioning significantly enhanced the antioxidant stress of cells, but there are still many problems that have not been clarified. The mechanism of preconditioning enhancing antioxidant stress is not completely clear, which signals are regulated by preconditioning. In addition, our study only explored the enhancement of antioxidant stress by oxidative stress preconditioning in vitro. When BMSCs are used for transplantation in vivo, there are many factors affecting its survival, such as post-injury ischemia, hypoxia microenvironment, and various inflammatory factors. Therefore, on how to improve the efficiency of BMSC transplantation, there are still many problems to be studied.

In conclusion, low-concentration H_2_O_2_ pretreatment can significantly increase the activity of SOD and CAT after oxidative stress injury, reduce the levels of ROS and MDA in cells, alleviate DNA and mitochondrial membrane damage, reduce cell apoptosis, and significantly promote the survival of BMSCs under oxidative stress conditions.

## Data Availability

The datasets during and/or analyzed during the current study available from the corresponding author on reasonable request.

## Abbreviations

BMSCs: Bone marrow mesenchymal stem cells
H_2_O_2_: Hydrogen peroxide
ROS: Reactive oxygen species
SOD: Superoxide dismutase
CAT: Catalase
MDA: Malondialdehyde
SPF: Specific pathogen-free
PBS: Phosphate-buffered saline
OD: Absorbance value
CPCs: Cardiac progenitor cells
